# Micronutrient Level Is Negatively Correlated with the Neutrophil-Lymphocyte Ratio in Patients with Severe COVID-19

**DOI:** 10.1155/2022/6498794

**Published:** 2022-01-31

**Authors:** Shengyu Zhou, Fayan Zhang, Fangfang Chen, Peng Li, Yujie He, Julong Wu, Liang Dong, Chunting Wang, Ximing Wang, Wei Zhang, Wenqing Sun, Lixia Yin, Rumin Zhang, Jintong Zhao, Baoxia Sun

**Affiliations:** ^1^Clinical Nursing Department, School of Nursing, Cheeloo College of Medicine, Shandong University, Jinan, China; ^2^Department of Respiratory and Critical Care Medicine, Qilu Hospital, Cheeloo College of Medicine, Shandong University, Jinan, China; ^3^College of Traditional Chinese Medicine, Shandong University of Traditional Chinese Medicine, Jinan, China; ^4^Health Inspection and Testing Institute, Shandong Provincial Center for Disease Control and Prevention, Jinan, China; ^5^Blood Center of Shandong Province, Jinan, China; ^6^Institute of Virology, Shandong Provincial Center for Disease Control and Prevention, Jinan, China; ^7^Department of Respiratory, Shandong Provincial Qianfoshan Hospital, Shandong University, Jinan, China; ^8^The First Affiliated Hospital of Shandong First Medical University, Shandong Institute of Respiratory Diseases, Jinan, China; ^9^Department of Critical Care Medicine, Shandong Provincial Hospital Affiliated to Shandong First Medical University, Jinan, China; ^10^Department of Radiology, Shandong Provincial Hospital Affiliated to Shandong First Medical University, Shandong University, Jinan, China; ^11^Department of Lung Disease, Affiliated Hospital of Shandong University of Traditional Chinese Medicine, Jinan, China; ^12^Department of Intensive Care Unit, Shandong Provincial Chest Hospital, Jinan, China; ^13^Department of Respiratory Medicine, Dezhou People's Hospital, Dezhou, China; ^14^Department of Critical Care Medicine, Zibo Central Hospital, Zibo, China; ^15^Infectious Department, Zaozhuang Municipal Hospital, Zaozhuang, China

## Abstract

**Aim:**

To explore the potential relationship between NLR and micronutrient deficiency in patients with severe COVID-19 infection.

**Methods:**

Sixteen patients were categorized into the mild group (mild COVID-19) and severe group (severe COVID-19) based on the guideline of the management of COVID-19. The lactate dehydrogenase (LDH); superoxide dismutase (SOD), the inflammatory markers (neutrophil lymphocyte ratio (NLR)), erythrocyte sedimentation rate (ESR), c-reactive protein (CRP), selenium (Se), iron (Fe), zinc (Zn), nickel (Ni), copper (Cu), chromium (Cr), cadmium (Cd), arsenic (As), and manganese (Mn) were measured in the blood.

**Results:**

Compared to the mild group, the NLR (*P* < 0.05) and the level of Se (*P* < 0.01), Fe (*P* < 0.05), and Zn (*P* < 0.05) were significantly decreased in the severe group. The level of Se, Fe, and Zn was significantly correlated to NLR levels. Furthermore, close positive correlation was found between NLR and severity of COVID-19.

**Conclusion:**

The micronutrient deficiency in the blood is associated with NLR in the severity of COVID-19 patients.

## 1. Introduction

It has been more than two years since the outbreak of COVID-19 in Wuhan, China. Despite effective measures such as wearing masks and social distancing, the cumulative number of confirmed cases and deaths worldwide continued to increase, reaching 242 million and 4.92 million, respectively, by 21 November 2021.

Research and medical management related to SARS-CoV-2 has evolved significantly in 2 years since the beginning of the epidemic. The respiratory tract, identified as the primary tissue for transmission of SARS-CoV-2, usually begins with influenza-like symptoms, but a certain percentage of patients may suffer a severe course of infection that can further lead to severe pneumonia, respiratory failure, and even death.

COVID-19 cases have confirmed that respiratory viral infections often lead to inflammatory progression [1]. COVID-19 patients with severe symptoms are prone to high inflammatory responses and even cytokine storms. Neutrophils and lymphocytes play important role in inflammation and are involved in the progression of COVID-19 [2, 3]. The neutrophil is the most abundant immune cell in the human body, which accounts for half of all leukocytes. As the front line of the immune system, neutrophils are essential in preventing infection. It also appears in a number of respiratory diseases, including influenza and SARS-CoV-19 [4]. Recently, neutrophil recruitment was revealed in the immune system triggered by SARS-CoV-2 infection. In addition, previous studies have suggested a positive correlation between the number of neutrophils and the severity of COVID-19; the more the neutrophils are, the worse the prognosis is [5, 6]. The lymphocyte is another type of leukocyte that plays a decisive role in maintaining immune homeostasis and defenses against external infections in the human body. A significant decline in lymphocytes was observed in COVID-19 patients. NLR is considered as an inflammatory marker and has been found to increase in a variety of conditions such as diabetes mellitus [7], thyroiditis [8], malignancy [9], irritable bowel disease [10], other thyroid conditions [11] and ulcerative colitis [12]. It has also been associated with COVID-19 infection, and many studies have shown that neutrophil-lymphocyte ratio (NLR) is a strong clinical indicator of COVID-19 severity [13].

Micronutrients, such as selenium and iron, are essential to keeping immune system homeostasis. Selenium is necessary for the proliferation and differentiation of the immune cells [14–16]. The deficiency of selenium could reduce humoral immunity activity and increase the respiratory tract infection risk [17]. Iron plays an important role in oxygen delivery by binding to hemoglobin. Iron deficiency will lead to immune system dysfunction and affect T-lymphocyte proliferation. Zinc could affect multiple aspects of the immune response. A previous study found that zinc deficiency could affect phagocytosis, intracellular killing, and neutrophil function [18]. Clinical studies confirmed that COVID-19 patients with severe symptoms tended to show lower selenium, iron, and zinc level [19–21].

The aim of this study was to explore the potential relationship between NLR and micronutrient deficiency in critically ill patients.

## 2. Materials and Methods

### 2.1. Study Population

This study was approved by the ethics committee of Qilu Hospital of Shandong University (ethical approval no.: KYLL-2020-GXB-001). All the participants in this study were enrolled between January 27 and May 18, 2020. Samples for the SARS-CoV-2 test were obtained from oropharyngeal swabs or nasopharyngeal swabs in all patients following “technical guidelines for COVID-19 laboratory testing, version 2.” Pathogen examination was performed using an ORF1ab/N Gene Nucleic acid detection kit (BioGerm, China) according to manufacturer's instructions.

All patients were divided into mild and severe groups based on the “Guideline on the Management of COVID-19” published by the National Health Commission of the People's Republic of China (version 8.0).

### 2.2. Measurement of Micronutrients

An electronic balance (HF-300, Japan, 2013000010) was used to weigh an appropriate amount of samples into the digestion tank, and 8 mL HNO_3_ was added for predigestion overnight. The microwave digestion instrument (America CEM, Mars6, instrument no. 2013000221) was used for digestion. Acid was driven to about 0.5 mL, which was carefully transferred to a 10 mL volumetric bottle, and the digestion tank was washed with a small amount of water. Transfer the lotion to a volumetric flask and add water to the volume. Iron was diluted 500 times with water in a 10 ml volumetric flask and then measured on a machine. At the same time, the blank test of the reagent was done.

Coupled plasma mass spectrometry (ICP-MS, iCAP RQ, ThermoFisher SCIENTIFIC, Hanna-Kunath-Str. 11, 28199 Bremen, Germany); working conditions: Rf power: 1350 W; sampling depth: 8.0 mm; plasma gas flow rate: 14.9 L/min; carrier gas flow: 1.2 L/min; diameter of sampling cone: 0.8 mm; diameter of the interception cone: 0.4 mm.

### 2.3. Standard Storage Solution

Barium, lead, cadmium, arsenic, aluminum, chromium, nickel, copper, vanadium, manganese, antimony, zinc, cobalt, iron, silver, beryllium, magnesium, thallium, and other 22 kinds of mixed standard storage solution: 20 *μ*g/mL, lot: M2-MeB657244.

The Se standard solution was diluted by 22 kinds of multielement mixed standard reserve solution to the concentration of 0 *μ*g/L, 1.00 *μ*g/L, 2.00 *μ*g/L, 5.00 *μ*g/L, 10.0 *μ*g/L, and 20.0 *μ*g/L, and the diluent was 1% HNO_3_.

The Ni standard solution was diluted by 22 kinds of multielement mixed standard reserve solution to the concentration of 0 *μ*g/L, 0.500 *μ*g/L, 1.00 *μ*g/L, 2.00 *μ*g/L, 5.00 *μ*g/L, and 10.0 *μ*g/L, and the diluent was 1% HNO_3_.

The standard Mn solution was diluted by 22 kinds of multielement mixed standard reserve solution to the concentration of 0 *μ*g/L, 0.500 *μ*g/L, 1.00 *μ*g/L, 2.00 *μ*g/L, 5.00 *μ*g/L, 10.0 *μ*g/L, and 20.0 *μ*g/L, and the diluent was 1% HNO_3_.

Fe standard use solution was diluted by 22 kinds of multielement mixed standard reserve solution to the concentration of 0 *μ*g/L, 10.0 *μ*g/L, 20.0 *μ*g/L, 50.0 *μ*g/L, 100 *μ*g/L, and 500 *μ*g/L, and the diluent was 1% HNO_3_.

Cu standard use solution was diluted by 22 kinds of multielement mixed standard reserve solution to the concentration of 0 *μ*g/L, 5.00 *μ*g/L, 10.0 *μ*g/L, 20.0 *μ*g/L, 50.0 *μ*g/L, and 100 *μ*g/L, and the diluent was 1% HNO_3_.

The standard use solution of Cr was diluted by 22 kinds of multielement mixed standard reserve solution to the concentration of 0 *μ*g/L, 0.100 *μ*g/L, 0.500 *μ*g/L, 1.00 *μ*g/L, 2.00 *μ*g/L, 5.00 *μ*g/L, and 10.0 *μ*g/L, and the diluent was 1% HNO_3_.

The standard use solution of Cd and As was diluted by 22 kinds of multielement mixed standard reserve solution to the concentration of 0 *μ*g/L, 0.100 *μ*g/L, 0.500 *μ*g/L, 1.00 *μ*g/L, 2.00 *μ*g/L, and 5.00 *μ*g/L, and the diluent was 1% HNO_3_.

### 2.4. Statistical Analysis

Results are expressed as mean ± SEM. Normality was tested for biological data. If the data were not normally distributed, then they were log-transformed. The data were analyzed using an unpaired *t*-test (GraphPad Prism 7, Graphpad, CV, USA). The correlation analysis was done by using Statistical Package for Social Sciences (SPSS) version 28 (IBM, USA), and Pearson's correlation analysis test was used in the analysis.

## 3. Results

### 3.1. Demographic Characteristics and Clinical Manifestations

**Figure 1:**
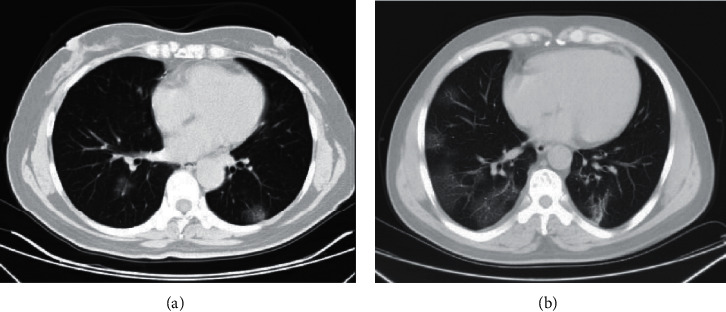
The representative CT images of COVID-19 patients. Mild patient (a). Severe patient (b).

Compared to the mild group, the body mass index (BMI) value in the severe group was significantly higher (*P*=0.0419, Table 1). The respiration rate (*P*=0.0047), temperature (*P*=0.0146), national early warning score (NEWS) (*P* < 0.0001), lactate dehydrogenase (LDH) (*P*=0.0025), and superoxide dismutase (SOD) (*P* < 0.0001) in the severe group were significantly higher than the mild group (Table 2). The oxygenation index in the severe group was dramatically lower than the mild group (*P* < 0.0001, Table 2). CT scan showed a patchy shadow in the lung from 3 patients in the mild group, but 8 patients in the severe group (Table 2). The images presented that patients in the severe group had more patchy shadows than the mild (Figure 1).

### 3.2. COVID-19 Severity Is Positively Correlated with the Level of Systemic Inflammation Markers and Neutrophil/Lymphocyte Ratio

**Figure 2:**
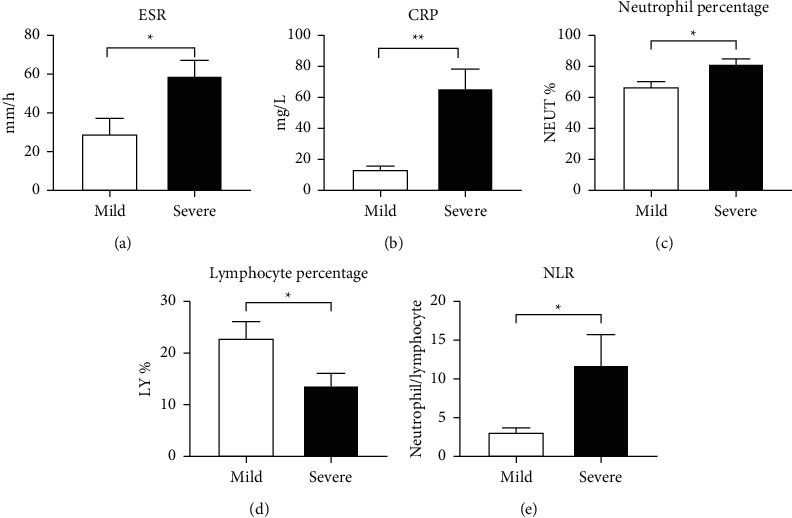
Systemic inflammation and leukocyte number measurement in blood from COVID-19 patients. ESR (a), CRP (b), neutrophil percentage (c), lymphocyte percentage (d), and NLR (e). Results are expressed as mean ± SEM and were analyzed by unpaired *t*-test. *n* = 8. ^*∗*^*P* < 0.05,  ^*∗∗*^*P* < 0.01. ESR: erythrocyte sedimentation rate; CRP: C-reactive protein. NEUT: neutrophil; LY: lymphocyte; NLR: neutrophil to lymphocyte ratio.

Compared to the mild group, the level of erythrocyte sedimentation rate (ESR) (*P*=0.0234, Figure 2(a)) and C-reactive protein (CRP) (*P*=0.0016, Figure 2(b)) in the severe group was significantly increasing. Neutrophil percentage in the whole blood was significantly higher in the severe group (*P*=0.0130 vs. mild group, Figure 2(c)), followed by the lower lymphocyte (*P*=0.0469 vs. mild group, Figure 2(d)). After calculation, the neutrophil to lymphocyte ratio was much higher in the severe group (*P*=0.0421 vs. mild group, Figure 2(e)).

### 3.3. The Level of Micronutrients Was Decreased in Severe COVID-19 Patients

**Figure 3:**
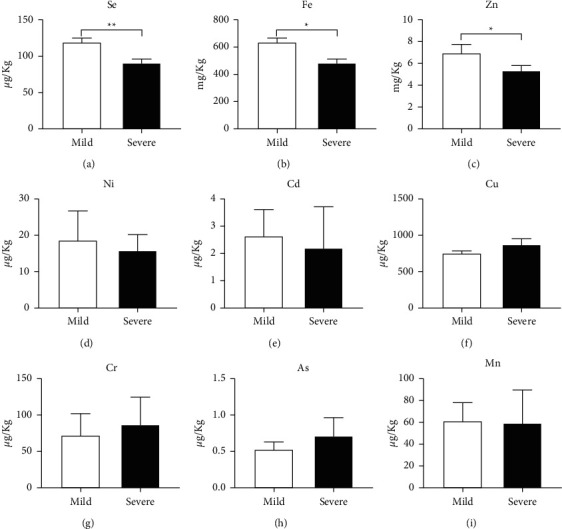
The micronutrient level in the blood from COVID-19 patients. Se (a), Fe (b), Zn (c), Ni (d), Cd (e), Cu (f), Cr (g), As (h), and Mn (i). Results are expressed as mean ± SEM and were analyzed by unpaired *t*-test. *n* = 8. ^*∗*^*P* < 0.05,  ^*∗∗*^*P* < 0.01. Se: selenium; Fe: iron; Zn: zinc; Ni: nickel; Cd: cadmium; Cu: copper; Cr: chromium; As: arsenic; Mn: manganese.

Compared to the mild group, the severe group had a significantly lower level of Se (*P*=0.0024, Figure 3(a)), Fe (*P*=0.0211, Figure 3(b)), and Zn (*P*=0.0930, Figure 3(c)). There was no significant difference between groups in Ni, Cd, Cu, Cr, As, and Mn (Figures 3(d)–3(i)).

### 3.4. Correlation Analysis

Correlation analysis showed that NLR had a significant and positive correlation with the severity of COVID-19 (Table 3).

The Se (*P* < 0.01), Fe (*P* < 0.05), and Zn (*P* < 0.05) had a significant correlation with NLR level after correlation analysis. Se, Fe, and Zn were all negatively correlated. There was no significant correlation between Ni, Cd, Cu, Cr, As, Mn, and NLR (Table 4).

## 4. Discussion

The results of this study showed that there were significant differences in Se, Fe, Zn levels, neutrophil count, and lymphocyte count between the two groups. Compared with the mild group, the levels of selenium, iron, zinc, and other trace elements in the severe group were significantly decreased. We found that the severity of COVID-19 was positively correlated with neutrophil count and negatively correlated with lymphocytes, as confirmed by another previous study. Selenium, iron, and zinc levels were negatively correlated with NLR levels.

Previous studies have reported that blood LDH levels can be used as an indicator of COVID-19 severity in large numbers of patients infected with SARS-CoV-2 in China. Higher LDH results in poorer prognosis in patients with SARS-CoV-2 infection [22]. This is consistent with our findings. LDH level was higher in the severe group. Oxidative stress is also involved in the progression of COVID-19 [23, 24]. SARS-CoV-2 infection may damage the immune system over time by disrupting the balance between oxidants and antioxidants in the body. The imbalance between the oxidative system and the synthesis of antioxidant enzymes produces oxidative stress and affects the pathogenesis of COVID-19 [23]. Elevated oxidative stress can activate the innate immune system and produce overreactions, such as cytokine storms, leading to greater lung damage and worsening clinical evolution [24]. SOD is an important antioxidant defense enzyme against oxidative stress, which is essential to maintain the balance of immune system. Our study showed that SOD levels were lower in the severe group with high oxidative stress.

Systemic inflammation is a marker of the progression of COVID-19 and can affect multiple organ function. In severe cases, an increased systemic inflammatory response can trigger a cytokine storm, leading to organ failure and even death. CRP is a sensitive indicator of acute systemic inflammatory response after viral infection and tissue injury. In previous studies, CRP levels were found to be positively correlated with the severity of COVID. The CRP level in the severe group was significantly higher than that in the nonsevere group [25]. We also found higher levels of C-reactive protein in the severe group. Erythrocyte sedimentation rate is another indicator of the level of chronic inflammation in the body. Previous studies have reported that ESR levels in patients with severe COVID-19 are significantly higher than those in the nonsevere group [25]. Our study confirmed similar results. One reason is that the severe group had higher levels of inflammation, which is consistent with CRP levels. Another possible explanation is that old age is usually accompanied by high ESR levels [26]. Also, age could accelerate the progression of COVID-19 by increasing oxidative damage [23]. The severe group in our study showed higher age.

Leukocytes are also involved in the systemic inflammation induced by the SARS-CoV-2 infection. Neutrophils are the most abundant white blood cells in the body and are vital to the immune system. Neutrophils tend for rapid chemotaxis to gather at the site of infection, play a defensive role, and inhibit and kill pathogenic microorganisms. A higher number of neutrophils indicates a protective effect at high levels of inflammation, but if neutrophils are too numerous, too widespread, and for too long, they can harm the lungs and cause pneumonia [27]. This is why the percentage of neutrophils in the severe group was significantly higher than that in the mild group. Lymphocytes, another type of white blood cell, are crucial in maintaining the homeostasis of the immune system. The number of lymphocytes was negatively correlated with the degree of inflammation. Previous studies have shown that lymphocytopenia is positively correlated with the severity of COVID-19 [3]. The same trend was observed in our study, with a significant decrease in the percentage of lymphocytes in the severe group. NLR consists of two types of white blood cells and reflects the dynamic balance between neutrophil and lymphocyte counts and systemic inflammation. Specifically, it represents the severity of inflammation. Our results suggest that NLR is a good predictor of disease severity for COVID-19. This is consistent with previous studies suggesting that the neutrophil-lymphocyte ratio can be used as a predictor of COVID-19 severity in South Asian and European populations [13, 28].

Previous studies have shown that micronutrient balance is essential for enhancing immune response and improving antiviral function [29]. Micronutrients, such as selenium and iron, have immunomodulatory functions and can influence susceptibility to various viral infections [30]. Selenium is essential for the proliferation and differentiation of immune cells [16]. Selenium deficiency can also affect neutrophil function. Animal models, including mice, rats, and cattle, have shown that neutrophils in these species lose their antipathogen function by disrupting the balance between producing enough free radicals to kill pathogens and avoiding producing too many free radicals themselves [31]. Previous clinical studies have found low selenium levels in patients discharged from intensive care units before treatment, and adequate selenium supplementation can enhance the immune response of patients with severe COVID-19 acute respiratory distress syndrome [21]. Iron deficiency can also cause neutrophil and lymphocyte dysfunction. After regulating inflammation, excess neutrophils should be safely removed by apoptosis. Iron deficiency destroys the balance between neutrophil proliferation and apoptosis and the apoptosis function. Finally, it leads to abnormal accumulation in the body [32]. Like selenium, iron deficiency disrupts the lymphocyte proliferation and reduces the circulating lymphocytes numbers in the blood by reducing translocation [30, 33]. In addition, previous studies have speculated that SARS-CoV-2 infection may cause iron deficiency by increasing circulating and tissue ferritin. The resulting hyperferriminemia causes high levels of oxidative stress induced by iron poisoning, inducing an inflammatory overreaction that leads to a severe outcome of COVID-19 [20]. Previous study reported that zinc ion is a micronutrient that owns antiviral effect against SARS-CoV-2 [15, 34]. Zinc ion was proved that it can inhibit the SARS-CoV-1 replication process by disrupting the activity of RNA-dependent RNA polymerase and modulate immune responses by inhibiting IL-6-stimulated intracellular signal transduction and inhibiting cytokine storms [35]. Conversely, the suppression of zinc ion could promote proinflammatory response [36]. These results suggest that micronutrient deficiencies may be associated with abnormal regulation of neutrophils and lymphocytes in COVID-19 patients and may influence NLR outcomes and disease severity. In the future, serum selenium, iron, and zinc contents may become important indicators to predict the severity of disease. Appropriate supplementation of selenium, iron, and zinc may contribute to the prognosis of the disease.

## 5. Limitations

Our study has limitations. First, the number of local patients in this study was low, and we were unable to include more local cases due to China's excellent disease prevention and control strategy. Second, micronutrient treatments were not included in the study. Despite these factors, our study may help inspire clinicians to better understand the relationship between NLR and micronutrients.

## 6. Conclusion

Serum selenium and iron deficiency are associated with neutrophil and lymphocyte counts and severity of COVID-19. Micronutrients should be considered in the treatment of COVID-19, especially in critically ill patients.

## Figures and Tables

**Table 1:** Demographic characteristics.

	Mild group	Severe group
BMI	23.9 ± 3.2	28.4 ± 4.7^*∗*^
Age	42.5 ± 6.93	51.9 ± 16.7
Gender (M/F)	5/3	4/4

Results are expressed as mean ± SEM and were analyzed by unpaired *t*-test. *n* = 8. ^*∗*^*P* < 0.05. BMI: body mass index.

**Table 2:** Clinical manifestations.

	Mild group	Severe group
Respiration rate	18.4 ± 0.9	28.6 ± 8.6^*∗∗*^
Temperature (°C)	37.0 ± 1.0	38.1 ± 0.6^*∗*^
NEWS	1.62 ± 0.744	9.4 ± 1.8^*∗∗*^
LDH	171 ± 31.72	354.8 ± 138.2^*∗∗*^
SOD	124.9 ± 27.9	94.6 ± 7.8^*∗∗*^
Oxygenation index	384 ± 22.8	166.5 ± 69.5^*∗∗*^
Blood pressure (systolic)	76.4 ± 9.7	74.9 ± 11.6
Blood pressure (diastolic)	123.5 ± 11.8	133.6 ± 15.8
CT patchy shadow	3	8

Results are expressed as mean ± SEM and were analyzed by unpaired *t*-test. *n* = 8. ^*∗*^*P* < 0.05,  ^*∗∗*^*P* < 0.01. NEWS: National Early Warning Score; LDH: lactate dehydrogenase; SOD: superoxide dismutase.

**Table 3:** Correlation of NLR with the severity of COVID-19.

Statistics	NLR
*R* value	0.595
*P* value	**0.025**

NLR: neutrophil and lymphocyte ratio.

**Table 4:** Correlation of Se, Fe, Zn, Ni, Cd, Cu, Cr, As, and Mn with NLR.

Statistics	Se	Fe	Zn	Ni	Cd	Cu	Cr	As	Mn
*R* value	−0.666	−0.562	−0.544	−0.172	−0.131	0.237	−0.043	0.169	−0.067
*P* value	**0.009**	**0.036**	**0.044**	0.556	0.655	0.414	0.883	0.563	0.821

Se: selenium; Fe: iron; Zn: zinc; Mn: manganese; Ni: nickel; Cd: cadmium; Cu: copper; Cr: chromium; As: arsenic. NLR: neutrophil to lymphocyte ratio.

## Data Availability

All data used to support this study are included within the article.
